# Association between dietary selenium intake and the prevalence of osteoporosis: a cross-sectional study

**DOI:** 10.1186/s12891-019-2958-5

**Published:** 2019-12-04

**Authors:** Yuqing Wang, Dongxing Xie, Jiatian Li, Huizhong Long, Jing Wu, Ziying Wu, Hongyi He, Haochen Wang, Tuo Yang, Yilun Wang

**Affiliations:** 1 Department of Orthopaedics, Xiangya Hospital, Central South University, Changsha, Hunan China; 2 Department of Epidemiology and Health Statistics, Xiangya School of Public Health, Central South University, Changsha, Hunan China; 3 Health Management Center, Xiangya Hospital, Central South University, Changsha, Hunan China; 4 Academic Rheumatology, Clinical Sciences Building, University of Nottingham, City Hospital, Nottingham, UK; 5 Arthritis Research UK Pain Centre, Nottingham, UK; 6 National Clinical Research Center of Geriatric Disorders, Xiangya Hospital, Central South University, Changsha, Hunan China

## Abstract

**Objective:**

To examine the correlation between dietary selenium (Se) intake and the prevalence of osteoporosis (OP) in the general middle-aged and older population in China.

**Methods:**

Data for analyses were collected from a population based cross-sectional study performed at the Xiangya Hospital Health Management Centre. Dietary Se intake was evaluated using a validated semi-quantitative food frequency questionnaire. OP was diagnosed on the basis of bone mineral density scans using a compact radiographic absorptiometry system. The correlation between dietary Se intake and the prevalence of OP was primarily examined by multivariable logistic regression.

**Results:**

This cross-sectional study included a total of 6267 subjects (mean age: 52.2 ± 7.4 years; 42% women), and the prevalence of OP among the included subjects was 9.6% (2.3% in men and 19.7% in women). Compared with the lowest quartile, the energy intake, age, gender and body mass index (BMI)-adjusted odds ratios of OP were 0.72 (95% confidence interval [CI] 0.55–0.94), 0.72 (95% CI 0.51–1.01) and 0.47 (95% CI 0.31–0.73) for the second, third and fourth quartiles of dietary Se intake, respectively (*P* for trend = 0.001). The results remained consistent in male and female subjects. Adjustment for additional potential confounders (i.e., smoking status, drinking status, physical activity level, nutritional supplements, diabetes, hypertension, fibre intake, and calcium intake) did not cause substantial changes to the results.

**Conclusions:**

In the middle-aged and older humans, participants with lower levels of dietary Se intake have a higher prevalence of OP in a dose-response manner.

## Introduction

Osteoporosis (OP) is a frequently-seen skeletal disease featured typically by micro-architectural deterioration and low bone mineral density (BMD), which are highly associated with the increase of bone fragility and susceptibility to fracture [1]. Nowadays, OP has been widely deemed a significant contributor to the social healthcare burden due to its high mortality, morbidity, and treatment expenses [2]. According to the data reported by five countries in Europe (i.e., the UK, France, Germany, Spain and Italy), the prevalence of OP in the male and female population ranged 7–8% and 30–33%, respectively [3]. In mainland China, a multi-center study yielded that the prevalence of OP was 6.46% in men and 29.13% in women who aged over 50 years [4]. The etiology of OP is multifactorial, among the already known etiologies, dietary factors are believed to have a great importance [5]. However, most of the existing studies have a focus on calcium intake [6–8], while other dietary factors, especially trace elements, which may also play a significant role in preventing OP, are seldom investigated [9].

Selenium (Se), a trace mineral element essential for human being, can regulate cellular processes by behaving as a component of Se-dependent antioxidant enzyme [10–12] that eliminates intracellular reactive oxygen species (ROS) [12, 13]. Therefore, deficiency in Se can lead to an increase in ROS levels, which has been considered as the proximal culprit in the pathogenesis of OP [14]. There were several human studies in the existing literature that investigated the association between Se status and BMD or osteoporotic hip fracture, a catastrophic outcome of OP, but the results are inconclusive. For example, with a sample of female participants aged 50–79 years, an earlier cross-sectional study suggested that dietary Se intake had no association with BMD [15]. However, the authors acknowledged a potential inclusion bias – most of their participants were women with normal BMD, which means the correlation between dietary Se intake and OP patients with low BMD, if any, might be missed [15]. In addition, another two studies reported a negative correlation between dietary Se intake and osteoporotic hip fracture [16, 17]. However, BMD was not directly measured in these studies [16, 17]. Meanwhile, two other studies indicated that plasma Se concentration is positively associated with BMD in healthy aging European men [18], also in healthy euthyroid postmenopausal women [19]. Another research indicated that serum Se deficiency was accompanied by ALOX12 variation, contributing to the development of OP [20]. But these studies concentrate on Se concentration in blood rather than dietary Se intake.

To fill in this knowledge gap, a large-sample cross-sectional study was conducted to elaborate the association between dietary Se intake and the prevalence of OP in a middle-aged and elderly Chinese population.

## Methods

### Study population

Chinese subjects who received health screening at the Department of Health Examination Centre of Xiangya Hospital, Central South University (located in Changsha of Hunan, China) within the time window from October 2013 to December 2015, were recruited for the present research. The study design remained consistent with some of our previously-published works [21–25]. To acquire the demographic information and health-related habits, registered nurses were engaged to interview all the participants during the physical examination by referring to a standard questionnaire.

Prior to research implementation, the study protocol had been reviewed and approved by the Ethics Committees on Research of Xiangya Hospital, Central South University (No. 201312459), and informed consent had been collected from all the participants after explaining the research content in verbal and written. For sample screening, the following inclusion criteria applied: (1) ≥40 years old; (2) the data of average consumption of specific food items and drinks during the last 12 months could be retrieved from the semi-quantitative food frequency questionnaire (SFFQ); (3) basic characteristics such as age, gender, body mass index (BMI), smoking status, alcohol drinking status, waist circumference, exercise intensity, and history of hypertension/diabetes were available; and (4) participants were not diagnosed with any other musculoskeletal disorders (e.g., rheumatoid arthritis, osteochondroma, and other bone tumors).

A total of 31,542 participants received routine physical examinations at aforementioned study center from October 2013 to December 2015, and 6471 of them were qualified by the inclusion criteria. Then, 200 participants were excluded due to the existence of other musculoskeletal disorders (e.g., rheumatoid arthritis, osteochondroma, and other bone tumors), and four participants were excluded due to the unavailability of dietary questionnaire data. Eventually, 6267 participants were included for final analysis.

### Dietary assessment

A validated SFFQ which was adopted in some of our previously-published studies [23, 26] was referred to for the assessment of dietary intake. The SFFQ survey was conducted twice for all participants, with at least a one-week interval in between, to comparatively evaluate their reproducibility based on the calculation of dietary Se intake. The correlation coefficient was 0.64 (*P* <  0.001). Then, a subsample (*n* = 173) was created by random selection from the study cohort, and was used to validate the SFFQ by comparing the result derived from SFFQ with that obtained from the 24-h dietary recall method over the same sample. The correlation coefficient for dietary Se intake was 0.47 (*P* <  0.001). The results of validation showed that the overall performance of SFFQ in the present study was consistent with previous works [27, 28].

In this SFFQ, a total of 63 food items were included based on the general dietary habit in Hunan province of China, with the intention to understand the participants’ frequency of consumption for each food item (i.e., never, once per month, 2–3 times per month, 1–3 times per week, 4–5 times per week, once per day, twice per day, or > twice per day) and average amount of consumption in each time (< 100 g, 100–200 g, 201–300 g, 301–400 g, 401–500 g, or > 500 g) during the previous year. The SFFQ consisted of 63 commonly consumed local food items, including the main sources of dietary Se, which included meat, fish, eggs, bread, cereals and milk [29]. Also included almost Se-free food sources, such as fruits and vegetables [30]. To facilitate the participants in making accurate choices, pictures of food items showing the standard weight were provided alongside the SFFQ. The compositions of macro nutrients and micro nutrients were calculated based on the Chinese Food Composition Table for all the included food items.

### Assessment of other exposures

The BMI was calculated based on the measurement of weight and height for each participant. The average frequency of physical activity (never, 1–2 times per week, 3–4 times per week or ≥ 5 times per week), average duration of each physical activity (< 30 min, 30–60 min, 1–2 h, or > 2 h), as well as the smoking and drinking status were all inquired and recorded during the interview. The fasting blood glucose (FBG) was detected by the Beckman Coulter AU 5800 (Beckman Coulter Inc., Brea, CA, US), and a participant would be diagnosed of diabetes if his/her FBG ≥ 7.0 mmol/L or if he/she was undergoing any anti-diabetic treatment. The blood pressure was measured by an electronic sphygmomanometer, and a participant would be diagnosed of hypertension if his/her systolic blood pressure ≥ 140 mmHg or diastolic blood pressure ≥ 90 mmHg, or if he/she was using any anti-hypertensive drug.

### BMD assessment

The BMD was detected by a compact radiographic absorptiometry (RA) system called Alara MetriScan (Alara Inc., Fremont, CA, US), at the middle phalanges of the second to fourth fingers on the non-dominant hand. To guarantee the accuracy of measurement, all participants were requested to take off accessories from the hand before testing. The RA system would capture a high-resolution radio graphic image at an intensity expressed in arbitrary units (mineral mass/area) based on the mean value. Then, by referencing to a manufacturer-provided database, the T-score, which was used to compare the measured BMD of a participant with the average BMD of young, healthy subjects of the same gender, would be computed [31–33]. The peripheral densitometry system used in this study was characterized by high portability, low cost, and low X-ray dose (< 0.02 μSV/test). Based on the collected measurements, the participants were classified in accordance with the recommendations specified by the World Health Organization. Specifically, the BMD level within 1 standard deviation (SD) vs. a young, healthy adult is regarded as normal; the BMD level ranged from 1 to 2.5 SD below a young, healthy adult is regarded as osteopenia; and the BMD level equal to or 2.5 SD below a young, healthy adult is regarded as OP [34]. Participants classified as normal and osteopenia were both regarded as non-OP.

### Statistical analysis

All the continuous data was presented as means ± standard deviations, and the differences were assessed by the one-way analysis of variance (data of normal distribution) or the Kruskal-Wallis H test (data of non-normal distributions). All the categorical data was presented as percentages, and the differences were assessed by the Pearson Chi-square test. Dietary Se intake was categorized, on the basis of quartile distribution of the study population, into four categories: ≤29.2 μg/day, 29.3–39.8 μg/day, 39.9–51.8 μg/day, and ≥ 51.9 μg/day. The odds ratio (OR) with 95% confidence interval (CI) was calculated for all the quartiles of Se intake, and the lowest quartile was considered as the reference. A total of three models were created for multivariable analysis: the first model targeted on the dietary energy intake (quartiles); the second model further incorporated the factors of age (40–49, 50–59, 60–69, ≥ 70 years), gender (male, female) and BMI (< 28, ≥ 28 kg/m^2^) on the basis of the first model; and the third model further incorporated the factors of smoking status (yes/no), alcohol drinking status (yes/no), physical activity intensity (continuous), nutritional supplements (yes/no), hypertension (yes/no), diabetes (yes/no), dietary calcium intake (quartiles) and dietary fibre intake (quartiles) on the basis of the second model. Then, subgroup analysis of gender was performed. Subsequently, restricted cubic splines regression, with three knots (29.2 μg/day, 39.8 μg/day, 51.8 μg/day) defined by the quartile distribution of dietary Se was conducted to evaluate the dose-response relationship between dietary Se intake and the prevalence of OP [35, 36]. Statistical software SPSS 21.0 and STATA 11.0 were used for data analysis. *P* <  0.05 was equivalent to statistically significant.

## Results

A total of 6267 participants (3627 males, 2640 females) aged 40 years or older (average 52.2 ± 7.4 years old) entered the present study. The prevalence of OP in the entire group was 9.6% (2.3% in men and 19.7% in women). The essential features of the study sample on the basis of the OP status are shown in Table 1. We have observed significant differences between OP and non-OP subjects with regard to age, gender, smoking and drinking status, BMI, hypertension, physical activity level, nutrients supplementation, dietary calcium intake, dietary fibre intake, dietary energy intake, and dietary Se intake.

**Table 1:** Basic characteristics of the OP and non-OP population (*n* = 6267)

Basic characteristics	OP status		*P*
	OP population	Non-OP population	
Number	602	5665	–
Age (years)	59.0 ± 6.9	51.5 ± 7.1	< 0.001
40–49 (%)	9.1	43.7	
50–59 (%)	42.0	41.3	
60–69 (%)	42.9	13.4	
≥ 70 (%)	6.0	1.6	
Gender (female, %)	86.2	37.4	< 0.001
Smoking (%)	8.8	26.6	< 0.001
Drinking (%)	18.3	42.8	< 0.001
BMI (kg/m^2^)	23.2 ± 3.0	24.6 ± 3.2	< 0.001
Obesity (BMI ≥ 28 kg/m^2^, %)	6.6	14.0	< 0.001
Diabetes (%)	12.1	11.2	0.499
Hypertension (%)	38.2	31.3	0.001
Activity level (h/week)	2.7 ± 3.7	2.1 ± 3.3	0.007
Nutritional supplements (%)	47.3	34.4	< 0.001
Dietary calcium intake (mg/day)	443.4 ± 318.0	486.4 ± 325.0	< 0.001
Dietary fibre intake (g/day)	15.8 ± 12.4	18.0 ± 14.9	< 0.001
Dietary energy intake (Kcal/day)	1481.7 ± 776.0	1638.2 ± 750.7	< 0.001
Dietary selenium intake (μg/day)	39.1 ± 31.1	44.0 ± 23.3	< 0.001

*OP* osteoporosis, *BMI* body mass index

*P* values are for the test of difference between the OP population and non-OP population using one-way analysis of variance in case of normally distributed continuous variables, Kruskal-Wallis H test in case of non-normally distributed continuous variables and Pearson Chi-square test in case of categorical variables

**Fig. 1:**
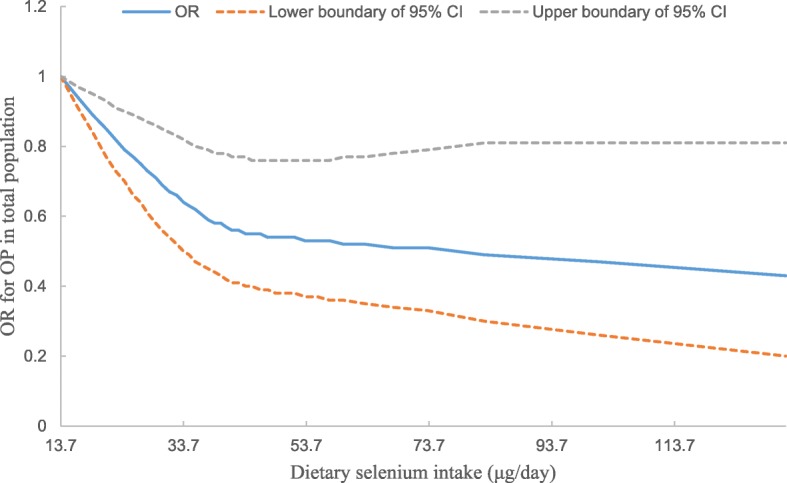
Dose-response relationship between dietary selenium intake and the odds ratio for osteoporosis in the total population (*n* = 6267). *OP* osteoporosis, *CI* confidence interval, *OR* odds ratio

The correlation between dietary Se intake and the prevalence of OP is demonstrated in Table 2. As illustrated in Model 1, after adjustment for energy intake, the ORs (95% CIs) of OP were 0.78 (95% CI: 0.61–0.99), 0.76 (95% CI: 0.56–1.01), 0.51 (95% CI: 0.35–0.75) for the second, third, and fourth dietary Se quartiles, respectively (*P* for trend = 0.001), in comparison with the lowest quartile. After further adjustment for age, gender and BMI in Model 2, the correlation between dietary Se intake and the prevalence of OP remained negative (*P* for trend = 0.001). The multivariable-adjusted ORs (95% CIs) for the prevalence of OP in the second, third and fourth dietary Se quartiles were 0.72 (95% CI: 0.55–0.95), 0.73 (95% CI: 0.51–1.03), 0.48 (95% CI: 0.31–0.75), respectively (*P* for trend = 0.002). Similar results were obtained separately for male and female subjects. Furthermore, as can be seen from Fig. 1, a negative correlation between dietary Se intake and the OR for OP was observed, with a dose-response relationship.

**Table 2:** Association between dietary selenium intake and the prevalence of OP (*n* = 6267)

	Quartiles of dietary selenium intake (μg/day)				*P* for trend
	Q1 (≤ 29.2)	Q2 (29.3–39.8)	Q3 (39.9–51.8)	Q4 (≥ 51.9)	
Median selenium intake (μg/day)	22.8	34.8	45.0	63.4	–
Total					
Model 1 (95% CI)	1.00 (Ref.)	0.78 (0.61, 0.99)	0.76 (0.56, 1.01)	0.51 (0.35, 0.75)	0.001
Model 2 (95% CI)	1.00 (Ref.)	0.72 (0.55, 0.94)	0.72 (0.51, 1.01)	0.47 (0.31, 0.73)	0.001
Model 3 (95% CI)	1.00 (Ref.)	0.72 (0.55, 0.95)	0.73 (0.51, 1.03)	0.48 (0.31, 0.75)	0.002
Male					
Model 1 (95% CI)	1.00 (Ref.)	0.33 (0.16, 0.66)	0.38 (0.19, 0.77)	0.20 (0.08, 0.46)	0.001
Model 2 (95% CI)	1.00 (Ref.)	0.33 (0.16, 0.68)	0.41 (0.20, 0.83)	0.22 (0.09, 0.52)	0.003
Model 3 (95% CI)	1.00 (Ref.)	0.35 (0.17, 0.71)	0.42 (0.20, 0.89)	0.25 (0.10, 0.61)	0.010
Female					
Model 1 (95% CI)	1.00 (Ref.)	0.77 (0.59, 1.00)	0.74 (0.52, 1.05)	0.53 (0.34, 0.84)	0.008
Model 2 (95% CI)	1.00 (Ref.)	0.83 (0.62, 1.11)	0.80 (0.54, 1.19)	0.54 (0.33, 0.90)	0.018
Model 3 (95% CI)	1.00 (Ref.)	0.82 (0.60, 1.10)	0.79 (0.53, 1.19)	0.53 (0.32, 0.89)	0.018

*Ref*. reference group, *OR* odds ratio, *CI* confidence interval

Model 1 included dietary energy intake (quartiles)

Model 2 included age (40–49, 50–59, 60–69, ≥ 70 years), gender (male, female), BMI (< 28, ≥ 28 kg/m^2^), and energy intake (quartiles) (age, BMI and energy intake for the gender subgroup)

Model 3 added smoking status (yes/no), drinking status (yes/no), activity level (continuous data), nutritional supplements (yes/no), diabetes (yes/no), hypertension (yes/no), dietary fibre intake (quartiles), and dietary calcium intake (quartiles) on the basis of model 2

## Discussion

In the present study, a negative association between dietary Se intake and the prevalence of OP was identified independent of major confounders. This correlation remained consistent across male and female subjects, and exhibited a dose-response relationship manner. The findings of our study may give a hint of the pathogenesis of OP, and future studies of dietary intake, including Se supplementary intake, on the risk of OP are warranted.

Several observational research works have investigated the association between dietary Se intake and BMD or osteoporotic hip fracture. For example, an earlier cross-sectional study by Wolf et al. indicated that dietary Se intake was not correlated with BMD [15]. Se intake of participants enrolled in this study was 94.1 μg/day, and calcium intake of participants enrolled in this study was 900.1 mg/day. Pedrera-Zamorano et al. [37] observed that elevated Se intake negatively affected BMD in postmenopausal female subjects aged over 51, but only if their calcium intake was below 800 mg/day at the same time. As long as the calcium intake exceeded 800 mg/day, Se intake no longer appeared to affect BMD. Besides, Se intake in this study was 95.5 μg/day. Considering that Se intake of participants enrolled in both studies were beyond Se intake in our population (43.5 μg/day), the variation in Se intake in both the studies might be insufficient to reveal any association. There were three studies [16, 17, 38] investigating the association between dietary Se intake and the risk of osteoporotic hip fracture, but none of them concerned the value of BMD, hence subjects of fracture which were probably not caused by OP might also be enrolled into the studies. Besides, two of these three studies [16, 38] focused on the smoking population, while our study concentrates on the general population. Given that smoking is not the only cause of oxidative stress, which has been considered as the critical mechanism of OP [14], the association between dietary Se intake and osteoporotic hip fracture in the general population is still inconclusive.

Several mechanisms linking Se with OP have been postulated. First, it has been reported that interleukin-6 (IL-6) and some other cytokines play a significant role in the pathogenesis of OP [39]. Se can exert anti-inflammatory actions, mediated in part by the inhibitory effects on IL-6 and cytokine activities [40], suggesting a mechanism by which Se can regulate bone turnover and, in turn, have a protective effect on OP. Second, evidence has also shown that ROS plays an important role in the development of OP [14]. Selenoproteins, which have been proved as the essential Se transporter to bones [41], can be expressed in both bone-resorbing osteoclasts and bone-forming osteoblasts, and eliminate ROS after being generated [42, 43]. Thus, a limiting threshold of Se is probably required for adequate selenoprotein-mediated antioxidant activity and bone maintenance [44]. Third, the Se-dependent iodothyronine deiodinases can control thyroid hormone turnover, and Se-dependent glutathione peroxidases are implicated in thyroid gland protection. Therefore, the lack of Se may increase the level of thyroid hormones in the blood [45], which may accelerate bone loss and lead to OP [46].

As the first study, to the best knowledge of the authors, directly relating dietary Se intake to OP in a general context, this cross-sectional study is characterized with several strengths. First, it demonstrated a potential role of appropriate dietary Se intake in preventing the development of OP. Second, by adjusting the multivariable model for a number of potential confounders, the reliability of the results was significantly improved. Third, with a relatively large sample size, this study was believed to have a low occasionality in the findings. Fourth, dietary Se intake in our study (43.5 μg/day) is similar to the dietary Se intake in European populations (40 μg/day) [47], thus, our findings may be generalizable to the European populations. However, dietary Se intake of American population (93 μg/day in women and 134 μg/day in men) [29, 47, 48] is higher than our population, so generalizing the findings to the American population may warrant further studies.

On the other hand, the limitations should be acknowledged as well. First, the causal relationship between dietary Se intake and the prevalence of OP was not addressed in the present study, so the conclusions should be reassured by further prospective studies. Second, the BMD was detected at the phalanges with a compact digital RA system in this study, while the gold standard for OP diagnosis is to measure BMD at the hip and spine using a dual-energy X-ray absorptiometry (DXA) [49, 50]. Unfortunately, DXA is a very costly diagnostic method requiring frequent calibrations, so it is still seldom used in developing countries. In fact, several cohorts have examined the efficacy of phalangeal RA while utilizing the same measuring system [31, 51, 52]. For example, Steven et al. [51] detected the BMD at the intermediate phalanges of the second to fourth fingers, the lumbar spine (L2-L4), and the total hip in 221 women (50–75 years) using RA (Alara Metriscan, Hayward, Calif., USA) and DXA (Hologic Inc., Waltham, Mass., USA), and concluded that the sensitivity of RA in identifying OP was 82.9% and its negative predictive value (i.e., the proportion of patients who have no OP, but received a negative test) was 90%, in comparison with DXA. This suggested that phalangeal RA could be used as an effective method for OP measurement. Third, deducing Se intake from SFFQ may be difficult, because Se content of foods is very variable [53], meanwhile, Se content can be affected by food preparation or cooking [54], and can vary differently by country and region [55]. However, SFFQ has been widely used in the previous studies as a tool of evaluating dietary Se intake, and has been proved to be a valid and reliable method to estimate Se intake [15, 38, 56, 57]. Fourth, because of no specific questionnaires being set, we did not exclude people who received hormone therapy, glucocorticoids and bisphosphonates in our final analysis. Considering that the proportion of hormone therapy, glucocorticoids, or bisphosphonates use in the general population in China is relatively low [58, 59], so the usage of these medicines may have a minor effect on our final results.

## Conclusion

In the middle-aged and elderly humans, participants with lower levels of dietary Se intake have a higher prevalence of OP in a dose-response manner.

## Data Availability

Data can be requested from the corresponding author.

## Abbreviations

BMD: bone mineral density
BMI: body mass index
CI: confidence interval
DXA: dual-energy X-ray absorptiometry
FBG: fasting blood glucose
OP: osteoporosis
OR: odds ratio
RA: radiographic absorptiometry
ROS: reactive oxygen species
SD: standard deviation
Se: selenium
SFFQ: semi-quantitative food frequency questionnaire
